# Muscular Adaptations to Concurrent Resistance Training and High-Intensity Interval Training in Adults with Type 2 Diabetes: A Pilot Study

**DOI:** 10.3390/ijerph20186746

**Published:** 2023-09-12

**Authors:** Giorgio Orlando, Jamie Pugh, Steve Faulkner, Stefano Balducci, Massimo Sacchetti, Giuseppe Pugliese, Ilenia Bazzucchi, Jonida Haxhi, Eduardo Martinez-Valdes, Deborah Falla, Konstantinos Manolopoulos, Myra A. Nimmo

**Affiliations:** 1 Research Centre for Musculoskeletal Science & Sports Medicine, Department of Life Sciences, Faculty of Science and Engineering, Manchester Metropolitan University, Manchester M15GD, UK; 2 College of Life and Environmental Sciences, University of Birmingham, Birmingham B152TT, UK; j.k.douglas-pugh@bham.ac.uk (J.P.); m.a.nimmo@bham.ac.uk (M.A.N.); 3 Centre for Sport and Exercise Medicine, School of Sport, Exercise and Health Sciences, Loughborough University, Loughborough LE113TU, UK; steve.faulkner@ntu.ac.uk; 4 Department of Engineering, School of Science and Technology, Nottingham Trent University, Nottingham NG14FQ, UK; 5 Diabetes Unit, Department of Clinical and Molecular Medicine, “La Sapienza” University, Sant’ Andrea University Hospital, 00185 Rome, Italy; s.balducci@hctdiabete.it (S.B.); giuseppe.puglise@uniroma1.it (G.P.); jonida.haxhi@uniroma1.it (J.H.); 6 Metabolic Fitness Association, 00015 Monterotondo, Italy; 7 Department of Human Movement and Sport Sciences, University of Rome “Foro Italico”, 00135 Rome, Italy; massimo.sacchetti@uniroma4.it (M.S.); ilenia.bazzucchi@uniroma4.it (I.B.); 8 School of Sport, Exercise and Rehabilitation Sciences, College of Life and Environmental Sciences, University of Birmingham, Birmingham B152TT, UK; e.a.martinezvaldes@bham.ac.uk (E.M.-V.); d.falla@bham.ac.uk (D.F.); 9 Institute of Metabolism and Systems Research, College of Medical and Dental Sciences, University of Birmingham, Birmingham B152TT, UK; k.manolopoulos@bham.ac.uk

**Keywords:** muscle dysfunction, type 2 diabetes, concurrent training, muscle quality and resistance training

## Abstract

This pilot study aimed to compare the effects of eight weeks of concurrent resistance training (RT) and high-intensity interval training (HIIT) vs. RT alone on muscle performance, mass and quality in adults with type 2 diabetes (T2DM). Twelve T2DM adults were randomly allocated to the RT + HIIT (n = 5) or RT (n = 7) group. Before and after training, maximal oxygen uptake (VO_2max_), muscle strength and power were evaluated by calorimetry, dynamometry and one-repetition maximum (1RM) test. Quadriceps muscle volume was determined by MRI, and muscle quality was estimated. After RT, VO_2max_ (+12%), knee muscle power (+20%), quadriceps muscle volume (+5.9%) and quality (leg extension, +65.4%; leg step-up, +223%) and 1RM at leg extension (+66.4%), leg step-up (+267%), lat pulldown (+60.9%) and chest press (+61.2%) significantly increased. The RT + HIIT group improved on VO_2max_ (+27%), muscle volume (+6%), muscle power (+9%) and 1RM at lat pulldown (+47%). No other differences were detected. Among groups, changes in muscle quality at leg step-up and leg extension and VO_2max_ were significantly different. The combination of RT and HIIT effectively improves muscle function and size and increases cardiorespiratory fitness in adults with T2DM. However, HIIT combined with RT may interfere with the development of muscle quality.

## 1. Introduction

Diabetes mellitus is the most common metabolic disorder worldwide, with a global prevalence in adults reaching 10.5% [1]. Recently, the International Diabetes Federation has estimated that more than 784 million people will have diabetes by 2045 [1]. These patients risk developing several complications affecting different body systems and organs, leading to loss of independence, poor quality of life and high mortality rates [2].

The muscular system is a typical target of type 2 diabetes (T2DM), responsible for a deterioration of its structure and function, becoming particularly marked with the development and progression of long-term complications [3]. These alterations are responsible for significant physical dysfunction, mobility limitations and a high risk of falling [4]. Functional deficits of skeletal muscle include lower muscle strength and power and greater muscle fatigability (defined as activity-induced reduction in strength) [5,6]. These defects are particularly marked on the lower body muscles and are attributable to a progressive loss of muscle mass and poor muscle quality (defined as strength per unit muscle volume) [7]. Glycation of the muscle fibre proteins (e.g., myosin and actin), mitochondrial dysfunction and microcirculation damage are the main mechanisms underlying muscle dysfunction in T2DM [3].

Exercise training is a widely recognised tool for treating and preventing T2DM and its long-term complications due to its multiple beneficial effects on cardiometabolic health and vascular, nervous and muscular systems [8]. Among the exercise-based solutions, resistance training (RT) is not only the optimal solution for mitigating muscle dysfunction and promoting muscle hypertrophy in people with diabetes, but it is also an effective tool for improving glycaemic control, bone mineral density, balance and gait performance [3,9].

Current recommendations for people with T2DM suggest performing concurrent aerobic training (AT) and RT because this combination elicits superior benefits for metabolic control compared with AT or RT alone [10,11]. The sequence of RT before AT promotes better glycaemic stability, resulting in better glucose management during the exercise session [12].

However, whether muscle adaptations in RT and AT differ from those induced by RT alone is still unclear because of the limited number of studies available and the lack of uniformity among the reported results [13,14]. One of the main barriers limiting T2DM patients engaging in exercise is the required time commitment [15]. An alternative to continuous aerobic exercise is high intensity interval training (HIIT), which has a shorter exercise volume meaning reduced time commitment. HIIT yields equivalent glycaemic benefits and provides greater improvement to VO_2max_ than continuous exercise, thus giving further clinical benefit beyond glycaemic control [16]. In addition, HIIT develops significant improvements in the structure and function of the vascular system compared with traditional continuous aerobic exercise [17], thus potentially enhancing muscle function. For these reasons, we decided to use a HIIT protocol as replacement for a continuous exercise protocol following RT in order to investigate the value of concurrent exercise as an intervention to counteract muscle complications in people with T2DM.

Therefore, we undertook this pilot study to compare the effects of eight weeks of concurrent RT and HIIT versus RT alone in improving upper and lower body muscle strength, muscle power, muscle size and quality in adults with T2DM. We hypothesised that adults with T2DM performing RT and HIIT or RT alone for eight weeks would have comparable improvements in muscle strength and power and similar increases in quadriceps muscle volume and quality.

## 2. Methods

### 2.1. Study Design and Population

Participants with T2DM were identified across two hospitals in the United Kingdom (Birmingham) and Italy (Rome) and recruited as a part of the Concurrent Training in Type 2 Diabetes (CONTRADIA) (ClinicalTrials.gov Identifier: NCT03278704) study. The CONTRADIA study was an open-label, parallel, randomised clinical trial comparing skeletal muscle adaptations (from muscle fibre to whole-muscle) to concurrent RT and HIIT with RT alone in people with T2DM. We focused on the whole-muscle adaptations to RT with and without HIIT for this analysis.

Seventeen male adults with T2DM were recruited across the two hospitals and randomly allocated to either the RT-only group (8 participants) or RT + HIIT group (11 participants). From the original cohort, one participant from the RT + HIIT group was excluded at screening and two participants from the RT + HIIT group dropped out of the study without giving a reason. Four participants (RT + HIIT = 3; RT = 1) were excluded because they missed more than 6 sessions during the exercise intervention. Thus, a total of 12 participants with T2DM were included in the analysis (RT + HIIT: 5 vs. RT: 7). Inclusion criteria included diagnosis as per the American Diabetes Association (ADA) criteria, aged 30–50 years, male sex, overweight/obese (27–40 kg/m^2^), waist circumference ≥ 94 cm, non-smoker and sedentary/physically inactive for at least 12 months. Exclusion criteria included treatments other than metformin, HbA_1c_ > 9% and any condition limiting or contraindicating physical activity, including diabetic peripheral neuropathy and coronary or peripheral artery disease.

Participants maintained their prescribed treatment and were instructed to maintain their regular diet and physical activity patterns but refrain from any formal exercise training throughout the study period. Participants performed an 8-week exercise training program, and anthropometric data, clinical measures, cardiorespiratory fitness, muscle performance and muscle volume were determined before and after the intervention.

This study was conducted in accordance with the Declaration of Helsinki and was approved by the Ethics Committee of Sant’Andrea Hospital (Italy) and the West Midlands—Black Country Research Ethics Committee (UK). All participants gave written informed consent.

### 2.2. Exercise Training

All exercise training sessions were carried out at three exercise centres across the UK and Italy under the supervision of exercise specialists and using the same training equipment (Technogym, Cesena, Italy). Participants undertook eight weeks of training, including a total of 24 exercise sessions, three sessions per week, with no more than two non-consecutive days without exercise. The exercise volume differed among the two exercise groups, with RT consisting of 150 min per week (50 min per session) and RT and HIIT consisting of 225 min of exercise per week (75 min per session, consisting of 50 min of RT plus 25 min of HIIT). Each exercise session included five minutes of warm-up and cool-down, respectively, which consisted of low-intensity continuous exercise of, for example, cycling or walking.

The RT group was asked to perform three sets of 10 repetitions at 70–80% of one maximum repetition test (1RM), with a fourth set to failure, for two upper and four lower body exercises. These included unilateral leg extensions and step-ups followed by the contralateral leg, bilateral lat pulldown, and chest press. The weight was increased when ≥12 repetitions were completed on two consecutive training sessions. Each set was separated by a 2 min passive recovery period. Upper and lower body exercises were interchanged to ensure sufficient recovery.

Concurrent training consisted of participants performing the same RT programme followed by 10 × 1 min bout of high intensity cycling designed to elicit 90% of the maximal heart rate (HR_max_), with each repetition separated by 1 min of low intensity cycling at 30–40% of the HR_max_. Training intensity was determined from maximal oxygen uptake (VO_2max_) testing, and the workload was increased when average heart rate across the ten intervals dropped below 85% HR_max_.

### 2.3. Assessment of Cardiovascular Risk Factors, Cardiorespiratory Fitness and Physical Activity Level

Cardiovascular risk factors and cardiorespiratory fitness were evaluated at baseline and after 3 days from the last exercise session. More specifically, anthropometric data, body composition and glycaemic control were assessed after an overnight fast (approximately 10 h). Body mass, height and BMI were evaluated by digital scale and stadiometer. Body composition (i.e., fat and fat-free mass) was measured by bioelectrical impedance using a TANITA body fat analyser (model no. TBF-410, TANITA Corporation, Tokyo, Japan) wearing light clothing, with shoes, jewellery and all items from pockets removed.

HbA1c and fasting plasma glucose were evaluated by high-performance liquid chromatography (Adams TMA1C HA-8160, Menarini Diagnostic, Florence, Italy) and standard analytical methods using the VITROS 5,1 FS Chemistry System (Ortho-Clinical Diagnostics, Inc., Raritan, NJ, USA), respectively. Waist circumference was measured at the narrowest part of the torso between the distal border of the lowest rib and the superior border of the iliac crest at the end of expiration [18].

Cardiorespiratory fitness was assessed by VO_2max_ using a continuous, ramped exercise test performed to volitional exhaustion on a cycle ergometer (Lode Excalibur, Groningen, The Netherlands) and an online breath-by-breath gas analysis system that was calibrated for each participant according to the manufacturer’s instructions (Rome: COSMED, Rome, Italy; Birmingham: Vyntus system, Mettawa, IL, USA). Participants were first familiarised with the exercise equipment before a 5-min warm-up cycle at a work rate of 30–50 W. The work rate then progressively increased to 16 W.min^−1^ until volitional exhaustion. HR and RPE were continuously recorded during the exercise test, and verbal encouragement was provided. Participants were instructed to maintain a pedal cadence between 80 and 100 r.min^−1^. The test was terminated when the participant met two or more of the following criteria for VO_2max_: failure to maintain a pedal cadence of 50 r.min^−1^, achievement of age predicted HR_max_, failure of VO_2_ or HR to increase with further increases in work rate, or a respiratory exchange ratio greater than 1.15. VO_2max_ was determined as the highest averaged value over an 11-breath moving average [19].

All participants were asked to wear an ActiGraph wGT3X-BT accelerometer (firmware 1.5.0, ActiGraph, Pensacola, FL, USA) on the dominant hip for seven days (excluding sleep and water-based activities) before the training. Physical activity levels were estimated in participants reporting ≥8 h wear time per day (between a capture window of 07:00–23:00) for ≥3 valid days [20]. Classification of time spent in sedentary behaviour (<100 counts.min^−1^), light-intensity physical activity (≥100–2019 counts.min^−1^) and moderate-to-vigorous physical activity (≥2020 counts.min^−1^) were defined using validation cut-points [21]. Daily averages were calculated as the individual sum of each minute of sedentary activity, light, and moderate-to-vigorous physical activity, divided by the number of valid days of wear.

### 2.4. Muscle Mass and Quality

A 1.5-Telsa MRI scanner (Signa HDe, General Electric Healthcare, Milwaukee, WI, USA) was used to image the quadriceps muscle (dominant limb) from the anterior superior iliac spine to the knee joint space with T1 gradient echo scanning sequence. Scans were performed at the baseline and 48 h after the last exercise session. The following parameters were used: matrix = 512 × 512; slice thickness = 5 mm; time to echo = 15 ms; and time to repetition = 450–850 ms. The anatomical cross-sectional area of vastus lateralis, vastus intermedius, vastus medialis and rectus femoris was analysed and outlined in every fifth image (25 mm), starting from the most proximal image in which the muscle appeared. Visible intramuscular fat, blood vessels and connective tissue were omitted [22]. Cross-sectional areas were then multiplied by the scan thickness and summed to provide individual muscle volume. Total quadriceps volume (cm^3^) was calculated as the sum of individual muscle volumes. Osirix software (version 8, Pixmeo, Geneva, Switzerland) was used to analyse the MRI images. Finally, muscle quality was calculated by dividing leg 1RM strength (i.e., leg extension and leg step-up) in kg with quadriceps muscle volume (cm^3^) as measured by MRI [23].

### 2.5. Muscle Performance

On a separate appointment (i.e., baseline and after 3 days from the last exercise session), participants were asked to perform isometric and isokinetic contractions of the knee extensor muscles (dominant limb) on an isokinetic dynamometer (Rome: Kin-Com, Chattanooga, TN, USA; Birmingham: CON-TREX MJ, PHYSIOMED, Regensdorf, Switzerland). They were familiarised with the testing procedures prior to data collection. Participants were tested seated with a back-support at the waist, thigh, and chest. The lateral femoral epicondyle of the knee was aligned to the axis of rotation of the lever arm and secured with resistance pads applied to the ankle and lever arm of the dynamometer.

The isometric task consisted of rapidly increasing the force exerted by knee extensors to a maximum and maintaining this level of intensity for 2–3 s. Knee and hip joints were set at a 90° angle (180°, full extension), and hips were strapped to minimise extraneous movement. Three maximal contractions were performed, separated by 3 min of rest to recover from fatigue. The greatest isometric force achieved was used in our analysis.

After a 10-min rest-stop, maximal concentric contractions of the knee were performed at angular velocities of 180° s^−1^. Participants were requested to extend the knee as strongly as possible with a range of motion from 80 to 170°. At least three, but no more than six, maximal efforts were allowed to produce three overlying curves, and the mean maximal torque production was recorded. Isokinetic power (W) was then calculated as a product of the peak torque (Nm) and angular velocity (rad/s). In each trial, strong verbal encouragement was given to the participants.

Upper and lower body muscle strength was measured by 1RM at least 72 h after the dynamometry measurements. 1RM was tested at leg extension, leg step-up, bilateral lat pulldown and chest press. Participants warmed up performing six repetitions at 40% of the estimated 1RM and then four repetitions at 60% of the estimated 1 RM. Thereafter, the weight was increased until a failed attempt occurred.

Maximal strength was recorded as the maximal weight lifted in one full range of motion, and the 1-RM was determined after no more than five attempts, with a four-minute recovery between attempts. An exercise specialist demonstrated lifting and breathing techniques for each exercise.

## 3. Statistical Analysis

Data are expressed as the mean ± SD for parametric variables, median and interquartile range (IQR) for non-parametric data, and percentages for categorical variables. All parameters were tested for normal distribution by visual inspection and using the Shapiro–Wilk test. For parametric data, paired t-tests were used to examine within-group differences, and independent t-tests were used to investigate the difference in the deltas between groups. If the assumption of normality was violated, the Wilcoxon signed-rank test was used to examine within-group differences. Effect size (ES) was calculated as Cohen’s d for deltas variables, and values of 0.2, 0.5 and 0.8 define small, medium and large effect sizes, respectively. Statistical significance was set at α = 0.05, and the SPSS statistical software package (version 28) was used to analyse all data.

## 4. Results

### 4.1. Baseline Measurements

The baseline characteristics of the participants are shown in Table 1. There was no difference among exercise groups regarding age, diabetes duration, BMI, fat-free mass and physical activity level. Similarly, muscle power and 1RM at leg step-up, chest press and lateral pulldown were not different among groups. Fat mass was significantly higher, and VO_2max_, isometric torque, 1RM and muscle quality at the leg extension were significantly lower in the RT group than in RT + HIIT. There was no difference in the quadriceps muscle volume among groups.

### 4.2. Follow-Up Measurements

On average, both RT and RT + HIIT groups completed 22 out of 24 (both RT and RT + HIIT, range = 18 to 24) sessions over the 8-week protocol (93 ± 3%), and no exercise-related adverse events, including hypoglycaemia, were reported in participants from both exercise groups. There were no changes in body composition, HbA_1C_ and fasting plasma glucose after training in both exercise groups. VO_2max_ increased significantly by 12% and 27% in both RT groups (Pre: 22 ± 0.9 mL/kg/min vs. Post: 25.6 ± 1.5 mL/kg/min, *p* = 0.034) and RT + HIIT (Pre: 29.7 ± 0.4 mL/kg/min vs. Post: 36.6 ± 1.8 mL/kg/min, *p* < 0.001), respectively.

In the RT group (Table 2), the 1RM at the leg extension (Pre: 19.8 ± 8.1 kg vs. Post: 32.1 ± 18.7 kg, *p* = 0.021), leg step-up (Pre: 20.7 ± 5.7 kg vs. Post: 72.7 ± 6.7 kg, *p* < 0.001), lat pull-down (Pre: 58.7 ± 18.3 kg vs. Post: 88 ± 23.9 kg, *p* = 0.005) and chest press (Pre: 37.7 ± 15.7 kg vs. Post: 58 ± 29.2 kg, *p* = 0.041) were increased significantly by 66.4%, 267%, 60.9% and 61.2%, respectively, after training. Muscle power (Pre: 328.4 ± 88.1 W vs. Post: 387 ± 94.2 W, *p* = 0.021) improved significantly by 20% after training, whereas only a tendency was seen for isometric torque (+25.4%, *p* = 0.060). Muscle volume of the quadriceps muscle significantly increased by 5.9% (Pre: 1.615 ± 328 cm^3^ vs. Post: 1.701 ± 304 cm^3^, *p* = 0.035), and muscle quality at leg extension (Pre: 0.012 ± 0.003 kg/cm^3^ vs. Post: 0.019 ± 0.009 kg/cm^3^, *p* = 0.026) and leg step-up (Pre: 0.011 ± 0.003 kg/cm^3^ vs. Post: 0.039 ± 0.005 kg/cm^3^, *p* = 0.002) improved by 65.4% and 223% respectively, after RT.

After RT + HIIT (Table 2), the 1RM lat pull down (Pre: 63.8 ± 7.4 kg vs. Post: 93.4 ± 11.5 kg; *p* = 0.003) and knee muscle power (Pre: 354.2 ± 40.3 W vs. Post: 383.9 ± 26.7 W, *p* = 0.037) was improved by 47% and 9%, respectively. No difference was detected for isometric torque. Muscle volume increased significantly by 6% (Pre: 1.691 ± 317.9 cm^3^ vs. Post: 1.778 ± 337.2 cm^3^, *p* = 0.004). Although there were large differences, no statistical difference was found for 1RM at leg extension (+43.6%, *p* = 0.068), leg step-up (+223%, *p* = 0.083) and chest press (+40.8%, *p* = 0.103) and muscle quality (leg extension, +33.2%, *p* = 0.080; leg step-up: +154%, *p* = 0.072).

**Figure 1:**
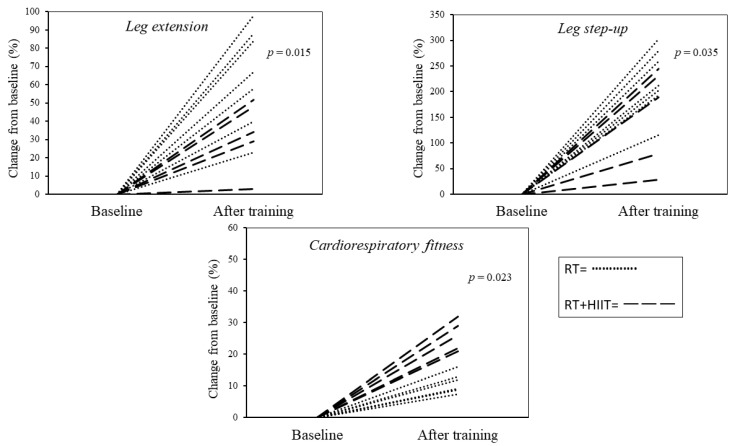
Cardiorespiratory and muscle quality changes for each participant after 8-weeks of exercise training.

Comparison among groups showed that across all parameters, only changes in VO_2max_ (RT: 12 ± 5.5% vs. RT + HIIT: 27 ± 4.6%, ES: 0.798, *p* = 0.023) and muscle quality at leg step-up (RT: 223 ± 53.1% vs. RT + HIIT: 154 ± 76.2%, ES: 0.432, *p* = 0.035) and leg extension (RT: 65.4 ± 27.2% vs. 33.2 ± 19.3%, ES: 0.468), *p* = 0.015) were significantly different. Figure 1 shows cardiorespiratory and muscle quality changes after exercise training for each participant in both groups.

## 5. Discussion

To the best of our knowledge, this study is the first randomised clinical trial to investigate structural and functional adaptations of skeletal muscle in response to a combination of strength and high-intensity aerobic exercises. The aim was to explore whether the latter (i.e., HIIT) interferes with muscle growth and muscle function improvements induced by RT in people with uncomplicated T2DM.

Our results suggest that relatively short duration of concurrent RT and HIIT are feasible and effective for improving muscle performance and promoting muscle hypertrophy in sedentary people with T2DM. Patients performing RT and HIIT reported a marked increase in upper and lower body muscle strength and modest increases in muscle power and mass. In addition, this modality was accompanied by a marked improvement in cardiorespiratory fitness, as indicated by a 27% increase in VO_2max_- (corresponding to a 6.8 mL/kg/min increase). These findings suggest RT combined with HIIT is a potent tool for treating muscle dysfunction of diabetes and for maximising cardiorespiratory benefits in uncomplicated patients with T2DM. As HIIT is characterised by a lower exercise volume than traditional continuous exercise, this modality should be more useful for diabetic patients because it partially overcomes the lack of time, which is one of the main barriers limiting the engagement in exercise of people with diabetes [15]. As such, this study and a previous one [24] show that this concurrent RT + HIIT model may offer an alternative strategy to feasibly increase the regular physical activity level in sedentary populations. Moreover, since a reduction of approximately 16% in mortality risk has been shown associated with every one metabolic equivalent of task (MET or approximately 3.5 mL/kg/min) increase in VO_2max_ [25]_,_ our results also support RT + HIIT as a powerful tool for reducing mortality in people with diabetes.

Our investigation points to adaptive outcomes that might occur when RT and HIIT are performed concurrently. Despite an insufficient study power that may have limited the capacity to detect differences in some functional parameters between groups, our findings indicate that concurrent RT and HIIT do not inhibit muscle hypertrophy but may interfere with muscle function adaptations in people with T2DM. Our data showed up to 62% additional improvement in muscle quality with RT compared with RT and HIIT. These data are in line partially with a recent meta-analysis [26] in healthy individuals, which indicates that the combination of HIIT and RT does not affect muscle hypertrophy, but it interferes, although minimally, with RT improvements. This discrepancy is unclear but could be explained by the differences across studies in the study population (diabetes vs. healthy), original physical activity level (sedentary vs. physically active) levels and exercise programme (e.g., exercise volume and sequence).

As the evidence on the effects of AT and RT on the muscular system in people with T2DM is limited, it is difficult to compare our results with previous studies. Indeed, only a few studies have compared muscle adaptations to RT with and without AT in T2DM. Furthermore, these focused exclusively on the effects of moderate-intensity AT on muscle performance and reported conflicting results [13,14]. Further studies are necessary to clarify whether the intensity of AT may modulate RT improvements and identify mechanisms underlying the interference effect of combining RT and HIIT.

Muscle improvements with RT align partially with the gains reported by several investigations exploring the effects of high intensity RT on whole-muscle function and muscle hypertrophy [3]. Indeed, our functional and structural improvements were more pronounced than those documented previously by several larger clinical trials that also show improvements in glycaemic control [23,27,28]. In particular, we found up to 267% improvement in muscle strength at lower limb, 20% higher knee muscle power and an increase of 6% in quadriceps muscle mass. These changes resulted in up to 223% higher muscle quality at the lower limb. The higher response in our sample could be related to the difference in RT protocol among studies and the clinical characteristics of the study population. Our protocol consisted of high-load RT focused particularly on leg muscles, whereas previous studies included a more generalised RT characterised by a progressive increase in the load until high intensity is obtained [3]. Our sample also included adults at the early stage of T2DM without complications and with good glycaemic control. Thus, it is possible that muscle adaptations were more marked in our group because of a lower impact of diabetes and a higher intensity of training on the muscular system. Altogether, our and previous findings [23,27,28] indicate that high-intensity RT is the optimal strategy for inducing multiple beneficial effects on the structure and function of the muscular system in people with T2DM, and these effects are particularly evident after a short period of training.

There are several limitations to the present study. First, our sample size was small, and this could affect the capacity to detect some statistical differences. A large, randomised, controlled clinical trial is needed to confirm the benefits of the interventions. Second, the baseline condition among the group differs in terms of cardiorespiratory fitness, fat mass, leg muscle strength and quality, which may indicate better physical fitness in people with RT + HIIT, and this could affect some statistical differences. However, participants were classified as physically inactive, sedentary and uncomplicated according to our inclusion criteria, and this strategy should mitigate the difference in the baseline measure between groups because of the same level of trainability, clinical condition and lifestyle across the participants. Third, as a pilot study, our sample included exclusively males in the early stage of diabetes in order to reduce individual/sex differences. However, this could limit the generalizability of our findings. Finally, we did not control the diet during the trial nor were the exercise groups matched for volume with the total exercise volume higher in RT + HIIT compared to RT. This could have increased the variability in some measurements and masked further interference. However, this study set out to examine the effects of a practical/realistic exercise model (RT + HIIT vs. RT), and therefore a prolonged RT-only training session would not have been appropriate.

## 6. Conclusions

In conclusion, this study shows that combined RT and HIIT increase whole body muscle strength, knee muscle power and quadriceps muscle mass and improve cardiorespiratory fitness in people with T2DM. RT combined with HIIT, however, interferes with the development of lower limb muscle quality. RT alone appears superior to the combination of RT and HIIT for optimising functional muscle improvements in people with T2DM.

## Figures and Tables

**Table 1:** Baseline characteristics of study participants.

Variables	RT	RT + HIIT	*p* Values
Number of cases	7	5	-
Age (years)	55.9 ± 1.3	54.2 ± 1.6	0.438
Diabetes duration (years)	4 (2; 12)	2(1.7; 11)	0.623
Body mass (kg)	108 (86.2; 115.3)	89.5 (85; 113)	0.122
BMI (kg/m^2^)	33.8 ± 3.6	31.8 ± 3.5	0.183
Fat mass (kg)	35 ± 8.7	26 ± 8.9	0.048
Fat-free mass (kg)	68.4 ± 8	71.2 ± 5.9	0.537
Waist circumference (cm)	113.7 ± 11.9	110.1 ± 14.2	0.646
HbA_1c_ (%)	6.6 ± 1	6.4 ± 1.5	0.837
FPG (mg/dl)	92.7 ± 29.8	114 ± 42.4	0.327
VO_2max_ (mL/kg/min)	22 ± 0.9	29.7 ± 0.4	<0.001
Leg step-up (Kg)	20.7 ± 5.7	16.5 ± 6.8	0.279
Leg extension (Kg)	19.8 ± 8.1	40.8 ± 31.5	0.022
Lat pulldown	58.7 ± 18.3	63.8 ± 7.4	0.358
Chest press (kg)	37.7 ± 15.7	42.4 ± 10.6	0.383
Muscle power (W)	328.4 ± 88.1	354.2 ± 40.3	0.559
Quadriceps volume (cm^3^)	1.615 ± 328	1.691 ± 317.9	0.707
Leg extension muscle quality (kg/cm^3^)	0.012 ± 0.003	0.026 ± 0.023	0.033
Leg step-up muscle quality (kg/cm^3^)	0.011 ± 0.003	0.010 ± 0.004	0.298
Physical activity (min.d)			0.670
Sedentary	531 ± 33	501 ± 42	0.535
LPA	293 ± 35	378 ± 49	0.129
MVPA	23 ± 6	25 ± 3	0.459

Data expressed as mean ± standard deviation or median and interquartile range as appropriate. Abbreviations: BMI = body mass index; HbA_1c_ = haemoglobin A_1c_; FPG = fasting plasma glucose; LPA = light-intensity physical activity; MVPA = moderate-to-vigorous intensity physical activity; RT = resistance training; RT + HIIT = resistance training and high-intensity interval training.

**Table 2:** Muscle structure and function before and after exercise training.

	RT			RT + HIIT		
Variables	Pre	Post	*p* Values	Pre	Post	*p* Values
Isometric torque (Nm)	143.2 ± 23.8	180.3 ± 54.8	0.060	209 ± 40.9	248.7 ± 39.3	0.204
Muscle power (W)	328.4 ± 88.1	387.3 ± 94.2	0.021	354.2 ± 40.3	383.9 ± 26.7	0.037
Leg step-up (kg)	20.7 ± 5.7	72.7 ± 6.7	<0.001	16.5 ± 6.8	54.5 ± 32.9	0.083
Leg extension (kg)	19.8 ± 8.1	32.1 ± 18.7	0.018	40.8 ± 31.5	50.4 ± 26.1	0.068
Lat pulldown (kg)	58.7 ± 18.3	88 ± 23.9	0.005	63.8 ± 7.4	93.4 ± 11.5	0.003
Chest press (kg)	37.7 ± 15.7	58 ± 29.2	0.041	42.4 ± 10.6	60.4 ± 25.2	0.103
Quadriceps volume (cm^3^)	1.615 ± 328	1.701 ± 304	0.035	1.691 ± 317.9	1.778 ± 337.2	0.004
Leg extension muscle quality (kg/cm^3^)	0.012 ± 0.003	0.019 ± 0.009	0.026	0.026 ± 0.023	0.030 ± 0.019	0.080
Leg step-up muscle quality (kg/cm^3^)	0.011 ± 0.003	0.039 ± 0.005	0.002	0.010 ± 0.004	0.030 ± 0.009	0.072

Data are expressed as the mean ± SD. Abbreviations: RT = resistance training; RT + HIIT = resistance training and high-intensity interval training.

## Data Availability

The data presented in this study are available on request from the corresponding author.
